# A phase I dose-escalation study of a biosimilar trastuzumab in Chinese metastasis breast cancer patients

**DOI:** 10.1186/s40064-015-1603-5

**Published:** 2015-12-22

**Authors:** Xinna Zhou, Jing Yu, Wenmiao Wang, Guohong Song, Xiaoli Wang, Jun Ren, Lijun Di, Xinghe Wang

**Affiliations:** Department of Medical Oncology, Key Laboratory of Carcinogenesis and Translational Research (Ministry of Education), Beijing Cancer Hospital & Institute, Peking University School of Oncology, 52 Fucheng Rd, Beijing, 100142 China; Department of Medical Oncology, Beijing Key Lab for Therapeutic Cancer Vaccines, Beijing Shijitan Hospital, Capital Medical University, 10 Tieyi Rd, Beijing, 100038 China; Phase I Clinical Center, Beijing Shijitan Hospital, Capital Medical University, 10 Tieyi Rd, Beijing, 100038 China

**Keywords:** Trastuzumab, Pharmacokinetics, HER2 overexpressed, Breast cancer

## Abstract

Trastuzumab has been widely used among the breast cancer patients with human epidermal growth factor receptor 2 (HER2) overexpression. The genetically engineered trastuzumab traded as Cipterbin® was developed in China since 2003. We have disclosed the phase I clinical trial data of safety, pharmacokinetic profile (PK) in patients with metastasis breast cancer. Subjects identified as HER2 strong positive received single intravenously doses of 100, 250 or 500 mg Cipterbin® in dose-escalation manner. The safety evaluations were recorded and plasma concentration profiles for the drug were analyzed. 27 Chinese metastatic breast cancer patients were enrolled in this study. Patients in each group of different dosage were well-tolerated. The most frequently drug-related adverse events were fever (59.3 %), transaminase increased (22.2 %), chills (18.5 %) and arrhythmia (18.5 %). Only one patient with severe adverse event was observed in 250 mg group revealing brachycardia. PK profile analysis showed that sera steady concentration could be reached in dose-proportional manner, except volume of distribution (V_d_) and clearance (CL), which reached peak values at 250 mg administration cohort. This genetically engineered HER2-target antibody had demonstrated the accepted safety with well-tolerated.

## Background

Trastuzumab is an IgG1 humanized monoclonal antibody that binds the extracellular domain of the human epidermal growth factor receptor 2 (HER2), disrupting the normal regulatory functions of HER2 signal pathways(Yarden and Sliwkowski 2001). Approximately 20–25 % breast patients are identified as HER2 overexpressed, which associated with high recurrence and shorten overall survival (OS) (Li and Li 2013; Kong et al. 2013).

Previous studies demonstrate that the addition of trastuzumab (Herceptin, F. Hoffmann-La Roche, Basel, Switzerland) to chemotherapy could significantly prolong the time of disease progression, duration of response and OS of metastatic breast cancer patients (Boekhout et al. 2011). Nowadays, the treatments of trastuzumab-containing regimens have been recognized as the standard of care in the treatment of HER2-overexpressed breast cancer patients. There are increasingly demands among the patients with HER2 overexpression who could be benefit on those standard treatments, but that is limited by the risk of drug shortages, which may greatly affect patient care and health care cost (Li et al. 2015), therefore biosimilars are pursued with great interest. The biosimilar product of trastuzumab (Cipterbin®) was developed by Shanghai CP Guojian Pharmaceutical Co. in China. Recombinant humanized anti-HER2 monoclonal antibody (rhuHER2mAb, Cipterbin®), a IgG1-κ antibody, with the complementarity determining region derived from a mouse anti-HER2 antibody and the rest parts from the human IgG1-κ antibody, which has the same binding sites and mechanism of action as Trastuzumab.

For this study, we have conducted a single institution, open labeled, phase I study. The primary objectives were designed to evaluate safety and toxicity of Cipterbin®. The secondary objective included pharmacokinetic evaluation of Cipterbin® in Chinese patients with metastatic breast cancer.

## Methods

### Patient eligibility

The patients were enrolled in Beijing Cancer Hospital from December 2004 to May 2005 under the circumstance of approved phase I clinical trial by China Food and Drug Administration of China. Enrolled patients were all women aged from 18–70 years with histological confirmed metastatic lesions Patients were required to have the evidence of overexpression HER2 (2+ or 3+) as determined by immonohistochemical staining(at least 10 % of carcinoma cells exhibited characteristic membrane staining). The main points of inclusive criteria was lacking of previously antitumor therapy within 4 weeks (6 weeks for mitomycin or nitrosureas). Eastern Cooperative Oncology Group (ECOG) performance status of less than 2; estimated life expectancy at least more than 3 months; adequate bone marrow, liver, and renal function were required. Patients with history of severe cardiac disease, central nervous system metastasis, active infections, pregnancy or lactation, previous therapy with similar monoclonal antibody, or concomitant use of any investigation agent were excluded. All patients signed the informed consent approved by the Ethics Committee of Beijing Cancer Hospital.

### Study design

This was a randomized, open label, dose-escalation study. As the study protocol designed 10 years ago, we had referred to few published literatures of transtuzumab (Herceptin®) and conducted this single arm phase I trial at that time. The three escalated dosage levels were 100, 250 and 500 mg, and each level assigned 9 patients, totally 27 patients enrolled in this study. Each patient was received single dose of Cipterbin® without any other anticancer therapy, and followed up until 70 days. During the study, peripheral blood tests and urine analyses were performed as the protocol required.

### Safety assessments

All patients underwent echocardiography during screening. Vital signs (blood pressure, pulse rate, temperature, and weight), physical examination (with particular attention to the cardiovascular system) and 12-lead electrocardiograms (ECG) were conducted throughout the study regularly (0.5, 2, 12, 24 h and every week during follow-up time of 70 days). Laboratory testing (hematology and biochemistry) were conducted at baseline, 24 h after first administration and every week during follow-up time. Ultrasound cardiograph used for left ventricular ejection fraction (LVEF) assessment was conducted at baseline and the end of study. The drug-related adverse events (AEs) were scored by National Cancer Institute Common Toxicity Criteria (CTCAE, Version 3.0). Drug-related AE was defined as the AE has possible or suspicious association with study treatment determined by the investigators.

### Pharmacokinetics

Blood samples for serum concentration of Cipterbin® (anti-Her2rhMAb) were collected before dosing, at 0.5, 2, 4, 8, 12, 24, 36 h and at 2, 3, 5, 7, 9, 11, 14, 21, 28, 35, 42, 49, 56, 63 days after first dosing. Serum levels of Cipterbin® were determined using a validated enzyme-linked immunosorbent assay (ELISA).

The Pharmacokinetics data were analyzed by Beijing Shijitan Hospital. All pharmacokinetic parameters were calculated by non-compartmental analysis based on serum concentration of Cipterbin® using Excel 2000 (Mircosoft, Redmond, Washington). SAS version 8.2 (SAS Institute Inc, Cary, North Carolina, USA) was used for statistical analyses, and all analyses were using actual time of sampling rather than scheduled times. Pharmacokinetic parameters were determined as follow: highest drug concentration observed in serum after administration (C_max_), area under the serum concentration versus time curve from time zero to last sampling (AUC_last_), area under the serum concentration versus time curve from time zero extrapolated to infinity (AUC_0–∞_), terminal half-life (t_1/2_), volume of distribution (V_d_) and clearance (CL). AUC was calculated using the linear trapezoidal rule. Terminal half-life was also evaluated following 0.693/K, when K was the terminal elimination rate constant.

## Results

### Patients characteristics

A total of 27 patients were enrolled in this study, and their characteristics were listed in Table 1. Patients aged from 28 to 67 years, and the median age was 51 years. 11 patients (40.7 %) had three or more sites of metastatic disease. Most patients had been pretreated before study entry, with a majority of patients (19/27) had received chemotherapies for metastatic diseases.

**Table 1:** Patient characteristics

Characteristic	Patients							
	Total (n = 27)		100 mg (n = 9)		250 mg (n = 9)		500 mg (n = 9)	
	No.	%	No.	%	No.	%	No.	%
Age, years								
Mean	51		51		53		49	
Range	28–67		40–67		36–64		28–63	
ECOG performance status								
0	18	66.7	8	88.9	5	55.6	5	55.6
1	9	33.3	1	11.1	4	44.4	4	44.4
Level of HER2/neu overexpression								
2+	8	29.6	3	33.3	2	22.2	3	33.3
3+	19	70.4	6	66.7	7	77.8	6	66.7
Receptor status								
Estrogen receptor-positive (n = 26)	14	53.8	4	44.4	3	37.5	7	77.7
Progesterone receptor-positive (n = 26)	9	34.6	3	33.3	2	25.0	4	44.4
Menopausal status								
Premenopausal	7	25.9	1	11.1	3	33.3	3	33.3
Postmenopausal	18	66.7	6	66.7	6	66.7	6	66.7
Perimenopausal	2	7.4	2	22.2	0		0	
No. of metastatic sites								
1	10	37.0	2	22.2	4	44.4	4	44.4
2	6	22.2	2	22.2	2	22.2	2	22.2
≥3	11	40.7	5	55.6	3	33.3	3	33.3
Dominant site of metastasis								
Bone	15	55.6	5	55.6	4	44.4	6	66.7
Lymph node	12	44.4	5	55.6	4	44.4	3	33.3
Viscera	10	37.0	5	55.6	2	22.2	3	33.3
No. of prior chemotherapy regimens for metastatic disease								
None	8	29.6	0		3	33.3	5	55.6
1	14	51.9	5	55.6	6	66.7	3	33.3
2	5	18.5	4	44.4	0		1	11.1
Prior hormonal therapy	13	48.1	8	88.9	2	22.2	3	33.3
Prior radiotherapy	15	55.6	4	44.4	6	66.7	5	55.6

### Safety evaluation

All 27 patients were eligible for assessment including tolerability and safety, and 25 of 27 (92.6 %) were encountered at least one frequency of drug-related AE in the study, as summarized in Table 2.

**Table 2:** Number of patients with drug-related adverse events

	Total (n = 27)		100 mg (n = 9)		250 mg (n = 9)		500 mg (n = 9)	
			Rang 1.35–1.89 mg/kg		Rang 3.65–6.25 mg/kg		Rang 6.41–11.11 mg/kg	
	Grade 1/2 No. (%)	Grade 3/4 No. (%)	Grade 1/2 No. (%)	Grade 3/4 No. (%)	Grade 1/2 No. (%)	Grade 3/4 No. (%)	Grade 1/2 No. (%)	Grade 3/4 No. (%)
Fever	16 (59.3)	0	5 (55.6)	0	3 (33.3)	0	8 (88.9)	0
Transit increased ALT/AST	6 (22.2)	0	1 (11.1)	0	3 (33.3)	0	2 (22.2)	0
Chills	5 (18.5)	0	2 (22.2)	0	2 (22.2)	0	1 (11.1)	0
Arrhythmia	5 (18.5)	1 (3.7)	2 (22.2)	0	2 (22.2)	1 (11.1)	0	0
Fatigue	4 (14.8)	0	1 (11.1)	0	2 (22.2)	0	1 (11.1)	0
Dyspnea	4 (14.8)	0	1 (11.1)	0	3 (33.3)	0	0	0
Arthralgia	4 (14.8)	0	2 (22.2)	0	2 (22.2)	0	0	0
Nausea/vomiting	3 (11.1)	0	1 (11.1)	0	2 (22.2)	0	0	0
Palpitation	3 (11.1)	0	1 (11.1)	0	2 (22.2)	0	0	0
Headache	2 (7.4)	0	2 (22.2)	0	0	0	0	0
Dry mouth	2 (7.4)	0	1 (11.1)	0	0	0	1 (11.1)	0
Rash	2 (7.4)	0	1 (11.1)	0	1 (11.1)	0	0	0

The most frequently drug-related AEs were fever (59.3 %), ALT/AST transient increased (22.2 %), chills (18.5 %), arrhythmia (18.5 %), without occurrence of mortality. In general, Cipterbin® was well-tolerated, the majority of the events were mild (grade 1/2) and transient, with the exception of one patient experienced serious adverse event of brachycardia in 250 mg group.

In this study, one patient suffered Grade 4 reversible sinus bradycardia. This patient was 39 years old and without history of any cardiac disease, after administrating of single dose of 250 mg (i.e. 6.25 mg/kg) Cipterbin®, she underwent serious sinus bradycardia (lowest heart rate 24/min) with syncope in the 13th day after drug administration. After treatment of promethazine and dexamethasone, the patient had been fully recovered. Other drug-related cardiac toxicities were mild and not intervention indicated, including three palpitation, three sinus bradycardia and one junctional premature beat. No significant decline in LVEF and no congestive heart failure occurred and there was no treatment-related death in this study.

### Pharmacokinetic analysis

**Fig. 1:**
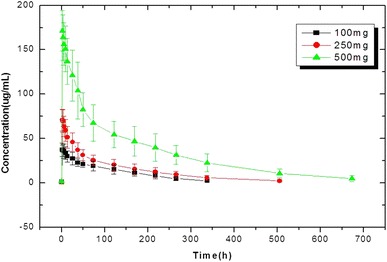
Serum concentration—time profiles. The figure showed the serum concentration—time of Cipterbin® at each does level, Cmax values occurred within 2 h after start of infusion, and subsequent rapid decline of serum concentration of Cipterbin® was followed by a slower elimination phase

The serum concentration—time profiles of Cipterbin® after first administration with each dose were shown in Fig. 1, and the pharmacokinetic properties of each dose were summarized in Table 3. As the mean serum concentration—time profiles, C_max_ values occurred within 2 h after start of infusion, and subsequent rapid decline of serum concentration of Cipterbin® was followed by a slower elimination phase. C_max_, AUC_0–672_ and AUC_0–∞_ were approximately dose-proportional manner. Terminal half-lives were increased in linear but less than dose proportional manner. V_d_ and CL were independent of dose. The peak value of V_d_ and CL happened at the dose of 250 mg.

**Table 3:** Pharmacokinetic parameters of Cipterbin® after single dose administration

Parameter	Unit	100 mg (n = 9)	250 mg (n = 9)	500 mg (n = 9)
AUC_0–672_	µg h/mL	4304 ± 1220	7368 ± 2142	22,386 ± 6774
AUC_0–∞_	µg h/mL	4510 ± 1214	7615 ± 2203	23,349 ± 7615
t_1/2_	h	81 ± 13	112 ± 20	144 ± 25
CL	mL kg/d	8.6 ± 1.7	13.5 ± 4.4	9.0 ± 2.8
V_d_	mL/kg	38.1 ± 6.2	74.0 ± 18.4	63.3 ± 18.9
C_max_	µg/mL	38.1 ± 7.4	72.5 ± 12.8	173.9 ± 23.8

Data are shown as mean ± SD

## Discussion

More and more accumulative data from the different clinical trials demonstrate the efficacy of trastuzumab among the patients with HER2 overexpression. Since the patent exclusivity rights for trastuzumab has expired in Europe and will lose in the United Stated in 2019, a number of biosimilars trastuzumab are undergoing the preclinical and clinical trials in different countries (Yin et al. 2014; Lopez-Morales et al. 2015; Wisman et al. 2014; Akbarzadeh-Sharbaf et al. 2012), and their pharmacokinetic features, efficacy and pharmacovigilance issues are arousing greater attention (Thill 2015; Cortes et al. 2014). We demonstrated the phase I data which finished over the past 10 years since the phase III data have just been achieved and the manufacturer approved to release the study data.

The comparison of the molecular structure between Herceptin® and Cipterbin® showed that the homology was similar in the domain of variable region of antigen binding, and the affinity and antigen binding profiles were similar *in vitro*. In this study, PK analysis data has displayed an approximately dose-proportional manner, except V_d_ and CL, which reached peak values at 250 mg administration cohort. Previous studies suggested that trastuzumab clearance related with baseline levels of circulating extracellular domain of the HER2 receptor or the number of metastatic sites in patients (Bruno et al. 2005).

Published evidence demonstrates that AEs of trastuzumab are mild and manageable when given as monotherapy or in combination with other treatments. The most commonly AEs are infusion-related reactions which including fever, chills, headache and other flu-like symptoms, often occurred within 24 h after infusion (Boekhout et al. 2011). 59.3 % patients in our study suffered from fever, which is higher than the patients treated with Herceptin in previous studies (up to 40 %)(Chung 2008; Cook-Bruns 2001), but these AEs were generally mild and transient, and the flu-like symptoms could be managed well with anti-inflammatory drugs when necessary.

Cardiac dysfunction is an important safety issue in trastuzumab treatment. From long-term cardiac safety data from large-scale randomized adjuvant trastuzumab trials, the incidence of symptomatic heart failure events was about 2 % in trasuzumab-treated with patients HER2-positive breast cancer. And the elder age (>50) and lower left ventricular ejection fraction (LVEF) (<50 %) at the baseline of trastuzumab treatment were closely associated with the higher rate of congestive heart failure (Russell et al. 2010). Our study design was intended to mitigate the risk of symptomatic heart failure by strictly selecting the patients has normal LVEF and carefully monitoring of cardiac function, and no significant decline in LVEF was observed in this study. Even though, the drug-related cardiotoxicities were still warranted. At present, echocardiographic measures and ECGs are used as the basic requirements, and some biomarkers have be comfirmed as the value predictors for precise evaluation of cardiotoxicities, such as the plasma level of N-terminal pro B type natriuretic peptide(NT-proBNP) and troponin (Perik et al. 2006; Cardinale et al. 2010).

In our study, 22.2 % patients occurred transient increase of serum aminotransferase levels at 24 h after infusion, and then these liver enzymes restored to normal within 14 days without medication, these cases resembled as rare report of trastuzumab-induced hepatotoxicity (Munoz et al. 2007; Srinivasan et al. 2008; Vucicevic et al. 2013). It should be noticed that the phase I study of a HER2 tyrosine kinase inhibitor CP-724 could elicit 66 % at least grade 1 hepatic toxicity (Guo et al. 2008). There was increasingly demand that the regular examination of liver function should be placed to avoid the asymptomatic appearance due to drug induced liver injury. Moreover molecular target reagents were capable of inducing the potential liver injury while much attention was paid in cardiotoxicities.

## Conclusions

We have primarily conducted this phase I trial and the major conclusion could be drawn that such biosimilar product was shown well-tolerated. We have admitted to say that although those data of phase I trial were collected in 10 years ago, the results still constitute the basis for the design of subsequent phase II and phase III clinical studies. The further efficacy and safety assessments should be warranted.
